# Accelerated Atherosclerosis and Cardiovascular Toxicity Induced by Radiotherapy in Breast Cancer

**DOI:** 10.3390/life13081631

**Published:** 2023-07-27

**Authors:** Miruna Florina Stefan, Catalin Gabriel Herghelegiu, Stefania Lucia Magda

**Affiliations:** 1 Department of Cardiology, University and Emergency Hospital, 050098 Bucharest, Romania; stefan_miruna@yahoo.com; 2 Institutul National Pentru Sanatatea Mamei si a Copilului “Alessandrescu Rusescu”, 020395 Bucharest, Romania; herghelegiu.cata@gmail.com; 3 Department of Cardiology and Cardiovascular Surgery, University of Medicine and Pharmacy Carol Davila, 020021 Bucharest, Romania

**Keywords:** cancer, radiotherapy, breast cancer, cardiovascular toxicity

## Abstract

The number of patients diagnosed with breast cancer and cardiovascular disease is continuously rising. Treatment options for breast cancer have greatly evolved, but radiotherapy (RT) still has a key role in it. Despite many advances in RT techniques, cardiotoxicity is one of the most important side effects. The new cardio-oncology guidelines recommend a baseline evaluation, risk stratification and follow-up of these patients. Cardiotoxicity induced by RT can be represented by almost all forms of cardiovascular disease, with atherosclerosis being the most frequent. An interdisciplinary team should manage these patients, in order to have maximum therapeutic effect and minimum cardiovascular toxicity. This review will summarize the current incidence, risk factors, mechanisms and follow-up of RT-induced cardiovascular toxicity.

## 1. Introduction

Cancer and cardiovascular diseases are among the most important causes of death worldwide. Breast cancer was the most frequently diagnosed cancer in 2020, accounting for 2.26 million new cases [1]. The life expectancy and overall survival of breast cancer improved significantly with the development of surgery, chemotherapy, radiotherapy (RT) and immunotherapy. The downside of this evolution is the cardiac toxicity that accompanies these therapies. Cardiotoxicity remains one of the most feared adverse reactions of cancer therapies, leading to increased morbidity and mortality both on the short term, as well as on the long term [2]. Damage appears to be related to dose, volume and technique of chest irradiation. Until now, hundreds of studies tried, without a definite result, to identify the safest dose to prevent cardiac adverse reactions and the most appropriate strategies to minimize RT-related cardiovascular disease are still debated [3].

While radiotherapy has definitely reduced local breast cancer relapses, it is associated, in some studies that evaluated long term effects of thoracic radiotherapy in general, to a significant increase in cardiovascular morbidity and mortality [4]. Consequently, clinicians face the challenging task of carefully weighing the advantages and disadvantages of radiotherapy when selecting appropriate treatment strategies. This is particularly critical since RT is employed as the sole therapeutic intervention in some cases and as an adjuvant therapy in others.

Of note, left- and right-sided breast cancer have been shown to have different morbidity and mortality, with the first one having a 1.3–1.6-fold higher risk of cardiovascular complications at a 10-year follow-up [5]. This might be due to the fact that the heart is located closer to the left breast, and thus more exposed to RT in this case. A large study showed that women with left-sided RT experienced an absolute risk increase of 73.9 cases of cardiac death per 100,000 person years (95%CI 41.6–113.9) compared with women receiving right-sided RT. Moreover, patients that received left-sided-RT had an increased risk of death from coronary heart disease (RR 1.23, 95%CI 1.07–1.41, *p* = 0.004), death from myocardial infarction (RR 1.35, 95%CI 1.12–1.63, *p* = 0.002), and myocardial infarction (RR 1.21, 95%CI 1.05–1.39, *p* = 0.007) [6]. However, newer studies, that analyzed cohorts of breast cancer patients exposed to modern RT techniques found no evidence that the laterality of breast cancer represents a risk factor for cardiovascular pathology after RT [7].

Before the 1970s, RT in breast cancer increased the risk of cardiovascular death by more than seven times [8]. Nowadays, preventive and protective measures evolved, but it still remains important to observe and describe the adverse cardiac effects of RT, in order to minimize them. Cardiovascular toxicity of RT should be carefully taken into consideration, especially when deciding between mastectomy and limited cancer resection.

## 2. Mechanism of Cardiovascular Toxicity

RT in general causes toxicity to all the layers of the heart, as well as its vascularization. At the center of the RT-induced myocardial injury is the endothelial cell damage [9]. The damage starts minutes after exposure to radiation and implies the activation of inflammation. This implies both microvascular, as well as macrovascular injury. Inflammation is a potent promotor of fibrosis [10]. Inflammation induces fibroblast cell recruitment, as well as the premature differentiation of fibroblasts, leukocyte recruitment, cytokine and free radical production [11]. The first phase of radiation-induced changes also implies oedema and the activation of the coagulation cascade [10]. The beginning of the fibrotic phase has been found to be after one month of radiation exposure [10]. Fibrosis can be found years after RT exposure in the walls of the coronary arteries, sometimes causing ostial lesions, as well as in all the layers of the heart, causing a wide spectrum of pathologies [5]. In particular, the amount of type I collagen increases proportionally to type III, which leads to a lower compliance of the tissues [12].

In the case of the arteries, inflammation causes macrophage activation and lipoprotein internalization, with the formation of new atherosclerotic plaques. The oxidation of lipoproteins in this inflammatory medium causes large necrotic cores that can at any time rupture, resulting in ischemic events [13]. Moreover, the media and adventitia are also more fibrous than in normal arteries [12]. The amount of fibrotic tissues increases progressively in the myocardium, which leads to a decrease in capillary density and increased myocardial stiffness, contributing to myocardial ischemia. This is the reason why diastolic dysfunction occurs first, followed later by systolic myocardial dysfunction [14]. Recent advances in technology allowed new discoveries in the complex mechanisms involved in the occurrence of RT-induced cardiotoxicity, which deserve a more detailed description, but will not be described in this paper, as its primary purpose remains the clinical impact of this pathology.

## 3. Definition of Cardiac Dysfunction and Classification

Clinically, cardiovascular dysfunction induced by any kind of RT can be classified as asymptomatic or symptomatic [2]. Depending on timing, cardiac dysfunction can be classified as acute (first two weeks), subacute, chronic or late chronic (after one year) [2]. Symptomatic cardiac dysfunction can further be classified into mild (mild heart failure symptoms, no intensification of medication is required), moderate (need for outpatient intensification of diuretic and heart failure medication), severe (requiring hospitalization) and very severe (requiring inotropic treatment, mechanical support or transplantation).

Moreover, asymptomatic cardiac dysfunction can further be classified as mild, moderate and severe. Severe asymptomatic cardiac dysfunction is defined as a newly observed left ventricle ejection fraction (LVEF) of <40%. Moderate asymptomatic cardiac dysfunction is defined by a new LVEF reduction by ≥10% to an LVEF of 40–49% or a new LVEF reduction by <10 percentage points to an LVEF of 40–49% and either new relative decline in global longitudinal strain (GLS) by >15% from baseline or a new rise in cardiac biomarkers (cTnI/T > 99th percentile, BNP ≥ 35 pg/mL, NtproBNP ≥ 125 pg/mL). Mild asymptomatic cardiac dysfunction is defined by an LVEF ≥ 50% and a new relative decline in GLS by >15% from baseline and/or a new rise in cardiac biomarkers [2].

According to the ASE/EACVI consensus, the decrease of LVEF by 10% to a value below 53% is considered as evidence of an existing cardiac dysfunction induced by cancer therapy [15]. The ASE/EACVI consensus defines that a relative percentage decrease of GLS by >15% from baseline is a clinically relevant evidence of subclinical left-ventricular dysfunction [15].

Radiation doses are expressed in Gray (Gy), which is the absorbed radiation. The worldwide accepted measure for RT induced cardiovascular toxicity is mean heart dose (MHD), because it takes into account not only the administered dose, but also the percent of the heart exposed to this radiation. Still, MHD is not perfect, as small pieces of the heart can be exposed to high radiation doses and the MHD in this cases would be small [2]. Nowadays, it is accepted that there is no safe dose of radiation. Therefore, efforts should be made to keep radiation exposure to the heart as low as reasonably achievable. Current group trials use a threshold of 3–5 Gy MHD to consider a treatment plan acceptable (e.g., NSABP-B51, Alliance-A221505). The risk of ischemic heart disease is increased by 4–16% per Gy of MHD. A linear relationship between MHD and myocardial infarction has also been observed [16]. Newer studies show that the utilization of individual cardiac radiation dose estimates in dose–response analyses is more appropriate for quantifying the risk of radiation-induced cardiac effects [17]. There is still a lack of comprehensive data on the risk of cardiac late effects in breast cancer patients who undergo contemporary radiation therapy, specifically regarding individual heart dosimetry.

## 4. Preventive Measures, Risk and Baseline Evaluation

Cancer and cardiovascular (CV) disease are closely linked by several crucial lifestyle risk factors, such as smoking, pollution, alcohol abuse, and a sedentary lifestyle. As a result, prioritizing the targeting of these factors should be a key strategy when seeking to prevent adverse cardiovascular events. Before starting any cancer treatment, a comprehensive baseline evaluation should be made and optimization of the treatment and pre-existing CV risk factors and/or CV disease is recommended [18]. A baseline electrocardiogram and a complete transthoracic echocardiogram should be obtained. Careful clinical examination should be made and an in-depth medical history should be recorded. Dosing of cardiac biomarkers, such as troponin and brain natriuretic peptides are, in some cases, useful. Before RT delivery, a review of available CT chest imaging for the presence of coronary and aortic calcifications to improve CV risk stratification is recommended [18].

The risk of developing CV disease after radiation is influenced by the dose of radiation per fraction, the volume of heart exposed to radiation, and the amount of coronary arteries that are in the radiation field (Table 1) [8].

Individuals categorized as high risk for cardiotoxicity following thoracic radiation therapy include patients who meet the following criteria: receiving a radiation dose exceeding 30 Gy with the heart included in the treatment area, undergoing both radiation therapy and anthracycline treatment, being younger in age, receiving high-dose radiation fractions surpassing 2 Gy per day, and possessing pre-existing cardiovascular risk factors. [19].

The recent cardio-oncology guidelines propose several recommendations for evaluating cardiovascular health prior to initiating cancer therapy. These recommendations include conducting a baseline assessment of cardiovascular risk and estimating the 10-year risk of fatal and non-fatal CV disease using either SCORE2 or SCORE2-OP. Additionally, it is advised to perform a baseline echocardiographic evaluation, preferably utilizing 3D methods to calculate LVEF, along with GLS if available. Lastly, an electrocardiogram (ECG) should also be obtained before initiating cancer treatment [2]. Moreover, at each visit, patients should be asked about classic cardiovascular symptoms and a basic physical examination should be performed [2]. CARDIOTOX (cardiovascular toxicity induced by cancer-related therapies) registry, showed that the increase in NT-proBNP or cTn at baseline was not associated with severe CV disease induced by cancer therapy in general [20].

In general, it is advisable to calculate the risk of cardiovascular events for cancer patients aged 40 years and above in order to effectively manage all cardiovascular risk factors. A newly developed predictive score, known as the CHEMO-RADIAT score, encompasses several factors including congestive heart failure, hypertension, elderly age, history of myocardial infarction or peripheral artery occlusive disease, obesity, renal failure, abnormal lipid profile, diabetes mellitus, left-breast irradiation, anthracycline dosage, and history of transient ischemic attack or stroke. This score enables comprehensive cardiovascular risk stratification among breast cancer survivors, providing clinicians with valuable information for making informed decisions regarding breast cancer treatment [21].

Primary prevention of the cardiac toxicity of radiotherapy mainly refers to technological advances. RT protocols should target the minimum volume required to the minimum dose needed to obtain the desired clinical benefit [2]. Implementing modifications in delivery techniques to minimize cardiac radiation exposure can significantly decrease the associated risk. Several methods employed to mitigate this risk include the utilization of intensity-modulated photon radiation therapy, adopting a prone position during treatment, employing deep-inspiration techniques, and incorporating image-guided radiation therapy such as three-dimensional conformal radiation therapy or volumetric modulated arc therapy. By incorporating these strategies, the potential for cardiac radiation exposure can be effectively reduced [2,22,23,24]. Reliable contouring also plays an essential role in achieving appropriate dosimetry. This may represent a real challenge for small structures when relying on lower-quality computed tomography [25].

Deep inspiration breathing hold (DIBH) has emerged as a widely embraced preventive measure for breast cancer patients undergoing radiotherapy (RT) due to its substantial reduction in radiation dose to the heart compared to free-breathing (FB) techniques. This technique has become increasingly prevalent in current practice [26]. DIBH has been shown to reduce 40–50% of the dose received by the heart [27]. Additionally, DEGRO provides specific dose constraints to minimize potential adverse cardiac effects. These recommendations include maintaining a mean heart dose below 2.5 Gy, a mean dose to the left ventricle (DmeanLV) below 3 Gy, limiting the volume of the left ventricle receiving ≥5 Gy (V5LV) to less than 17%, restricting the volume of the left ventricle receiving ≥23 Gy (V23LV) to less than 5%, ensuring a mean dose to the left descending artery (DmeanLAD) below 10 Gy, limiting the volume of the left descending artery receiving ≥30 Gy (V30LAD) to less than 2%, and constraining the volume of the left descending artery receiving ≥40 Gy (V40LAD) to less than 1%. Adhering to these guidelines helps minimize the potential for adverse cardiac effects [28,29]. A study that aimed to analyze real life data from patients with breast cancer treated in Germany with RT during 1998–2008 showed an important amount of variability in heart dose, with a mean heart dose of 3.7 Gy for left-sided breast cancer and 1.4 Gy for right-sided breast cancer and 66% of patients had a volume of 2 cm^3^ from the heart exposed to more than 40 Gy. Moreover, this study importantly highlighted the fact that when analyzing cardiac effects after RT, individual dosimetry is needed [30].

The recent study of Acevedo et al. proved the great advances made by modern RT techniques, showing in case of breast cancer patients a reduction in radiation dose from 13.3 Gy (used in the 1970s) to the present-day dose of 1.7 Gy. Moreover, this study investigated 10-year overall survival, which was estimated using a predictive tool, and the risk of CV events. Modern RT decreased 10-year breast cancer mortality by 4% but increased CV mortality by 0.2% [31]. Moreover, Hassan et al. investigated GLS and biomarkers for 70 women at 4 weeks and 2 months post-RT exposure, showing that modern proton-RT did not affect left-ventricle function and was not associated with an increase in biomarkers [32]. Some recent studies conducted in Germany examined the relationship between cardiac radiation exposure and the occurrence of cardiac late events in women diagnosed with breast cancer between 1998 and 2008. One of these studies found the average radiation dose to the heart for cases with left-sided breast cancer to be 4.27 Gy, while for cases with right-sided breast cancer, it was 1.64 Gy. In contrast to previous research, this study did not provide evidence linking radiation dose to the heart from 3D-conformal radiation therapy for breast cancer patients with an increased risk of cardiac events [17]. The contrast with older studies may be caused by the fact that the MHD in this study were considerably lower than those reported in previous studies and that previous studies mainly included patients treated before 2000. Other very important aspects when analyzing differences between studies lies in the definition for the cardiac event endpoint and the follow-up period.

No drug therapy has showed benefits in secondary prevention, but the control of all classic cardiovascular risk factors is highly recommended [2]. Still, statins should be considered as primary prevention in patients at high and very high risk for cardiac toxicity and beta blockers, and ACEi should be given to those considered at high risk to develop heart failure as a result of cancer therapy [2]. In the PRADA trial (prevention of cardiac dysfunction during adjuvant breast cancer therapy), concomitant treatment with candesartan attenuated the reduction in LVEF in women receiving treatment for breast cancer, whereas metoprolol attenuated the increase in troponins. However, it should be noted that the study examined adjuvant breast cancer therapies comprehensively, and it is not possible to draw conclusions specifically about the protective effects of this medication in RT alone [33].

Massanat et al. investigated the role of breast-tumor localization in the total amount of radiation received by the heart. It showed higher radiation exposure to the heart in patients with lower inferior quadrant tumors but reached significance only for V40 (which is the volume receiving 40% isodose) and maximum dose. Therefore, tumor localization should be taken into account when choosing RT as part of cancer management [34].

## 5. Radiotherapy-Induced Cardiovascular Toxicity

**Figure 1:**
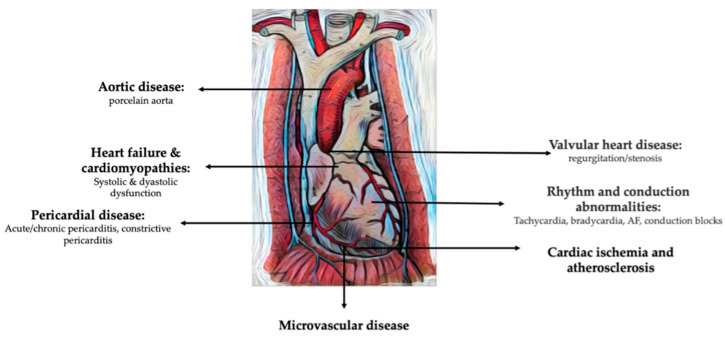
Cardiovascular pathologies induced by radiation therapy for breast cancer (modified and adapted from [35]).

Breast cancer survivors who undergo radiotherapy are at risk of developing a wide spectrum of cardiovascular pathologies, including cardiac ischemia, cardiac dysfunction, heart failure, valvular heart disease, arrhythmias, QT interval prolongation, and autonomic dysfunction (Figure 1) [2].

a. **Cardiac ischemia and atherosclerosis**

Coronary artery disease (CAD) is by far the most frequent form of cardiovascular toxicity of RT; some studies conducted in patients treated with thoracic RT for other kind of cancers reported an incidence as high as 85% [36]. A large meta-analysis that included 39 studies with breast cancer survivors revealed a higher risk of coronary disease and mortality during the first and second decade after RT. They found the dose of 5 Gy to the left ventricle as the most predictive for coronary events [37].

Any vascular bed exposed to RT is at increased risk of accelerated atherosclerosis [18]. This results in, among other effects, an early manifestation of coronary artery disease. The first signs of radiation-induced CAD were observed after the Hiroshima and Nagasaki atomic bombings. In this observed group, 10% died of CV disease [13,38].

In the case of the coronary arteries, the time between RT exposure and the development of CAD depends on the grade of atherosclerosis present before initiation of the treatment, exposure to classic CAD risk factors, as well as on the age of the patient at cancer diagnosis, the most affected being the younger ones [16]. Classic CV risk factors double the risk of a major coronary event [16]. CAD development also depends on the administered dose and the cardiac field that was exposed, many studies showing differences in CAD development between women that received RT for left versus right breast cancer. A 7.4–16.5% relative increase in rates of coronary events with each additional Gray has been observed [16,39]. Lai et al. evaluated coronary artery calcium (CAC), showing a higher risk CAC burden in the left-sided breast cancer patients compared to right-sided breast cancer patients exposed to RT [40]. Still, one recent study made on Asian cancer patients did not reveal a significant increase in the risk of ischemic heart disease-related mortality or overall mortality comparing left- vs. right-sided breast cancers in modern-era RT [41]. On the other hand, Tagami et al. showed that left-breast-cancer patients treated with RT have a significantly higher incidence of CAD compared with right-breast-cancer patients. Moreover, radiation doses are correlated with the incidence of CAD [42].

According to the BACCARAT study (breast cancer and cardiotoxicity induced by radiotherapy), the radiation doses administered to patients with right-sided breast cancer were significantly lower compared to those with left-sided breast cancer (*p* < 0.001), with the exception of the dose to the right coronary artery (RCA), which was higher in right-breast-cancer patients. The study also revealed that the mean dose to the heart and the mean dose to the left ventricle were significantly higher in patients who experienced CAC progression compared to those without CAC progression. Among the arteries assessed, the left anterior descending artery (LAD) was found to be the most exposed and affected. Non-zero CAC at baseline was identified as a major risk factor for CAC progression, along with traditional CAD risk factors and medication use. Therefore, patients with pre-existing coronary atherosclerosis and CAD risk factors are at a higher risk of accelerated CAD after exposure to radiotherapy. Additionally, the BACCARAT study demonstrated that subclinical left ventricle dysfunction, defined as a decrease in GLS greater than 10%, is associated with cardiac radiation doses. This suggests that left ventricle dosimetry could potentially assist in identifying high-risk populations susceptible to cardiac damage induced by radiotherapy [43]. The decrease in left ventricle strain was most significant in the endocardial layer. In other words, the endocardial layer-based longitudinal strain (LS) might be the most sensitive parameter to predict cardiac damage [44].

RT-induced atherosclerosis is characterized by long, diffuse and concentric lesions [45]. Optical coherence tomography (OCT) of these coronary lesions confirms high amounts of fibrin, lipids, and macrophages in these lesions [46]. Necropsy studies showed that the atherosclerotic plaques of people exposed to RT are more fibrous than of people not exposed to RT [47]. In addition to the accelerated atherosclerosis, the local inflammation provoked by RT increases intimal proliferation, platelet aggregation, activates the coagulation cascade and promotes clot formation. Moreover, free radicals cause DNA disruption, preventing replication and protein synthesis and thus contributing to fragile tissue formation. Therefore, when following-up their patients, clinicians should take into account that CAD could manifest as sudden cardiac death, and syncope, acute or chronic coronary syndromes.

In the development and localization of CAD, the radiation dose is an important predictive factor. One study showed that the dose–volume histogram was significantly higher in patients with left-sided breast cancer. Those patients had more perfusion abnormalities detected by perfusion SPECT, predominantly in the apical region and the anterolateral walls [48]. Scintigraphic defects can appear before electrocardiographic or classic echocardiographic changes; so, screening for silent ischemia should be taken into account.

A recent study showed that the dose received by the LAD correlated with adverse cardiac events. Minimizing the dose administered to the LAD should be of great importance in minimizing ischemic events in left-breast cancer survivors [49].

When treating thoracic RT-induced CAD, PCI should be performed using the implantation of a drug-eluting stent (studies showing no difference in cardiac mortality between patients with and without prior chest RT) [50], rather than using bare-metal stents or balloon angioplasty (increased risk of all-cause and CV mortality in patients exposed to RT) [4].

Surgical myocardial revascularization remains an option, but case selection is more difficult and should be applied only to particular cases. Among the difficulties encountered, there are the following: harder healing of the tissues exposed to RT, fragile native coronary arteries and the possibility of atherosclerosis or other inadequacy of the internal mammal arteries that have also been exposed to RT [51]. Studies show that restenosis of the grafts is more frequent than in the general population in people exposed to RT [52]. Another important aspect when considering surgical revascularization of the myocardium in thoracic cancer survivors is the fact that the thoracic aorta might be severely calcified, which would make aortic clamping for cardiopulmonary bypass difficult and dangerous (risk of aortic dissection or stroke) [52].

Hence, we consider that screening for CAD should be performed in all breast cancer survivors that have been exposed to RT. Functional stress testing (stress echocardiography, stress single-photon emission CT (SPECT)/positron emission tomography (PET), and stress CMRI) and CT imaging should be taken into account to detect CAD. Previous studies recommend functional imaging or CCTA, beginning at 5 years post RT [53].

One study that analyzed aortic distensibility after RT in breast cancer showed a significant decrease in aortic distensibility and increase in hs-CRP in the patients that were exposed to RT compared to the patients that were not. Importantly, the aortic distensibility was significantly related to age, systolic blood pressure, and RT dose, the aortic distensibility decreasing with higher radiation doses [54].

b. **Valvular Heart Disease**

Valvular heart disease is another frequently observed pathology after RT exposure, the incidence being as high as 26% after 10 years and 60% after 20 years [8]. Patients who have undergone RT display fibrotic changes in their heart valves that are similar to those found in other areas of the heart. This includes thickening and an increased concentration of collagen, which exceeds that of degenerated valves in other patient populations [8].

RT usually affects the left-sided valves, with aortic regurgitation being the most frequent valvular disease after radiation exposure, followed by aortic stenosis [13]. One trademark of patients exposed to mantle-RT is calcification of the aortomitral continuity, which can be observed at echocardiography [55]. In the case of the mitral valve, the base and middle portions of the anterior mitral valve leaflets are being affected, sparing the mitral valve tips and commissures, which makes it distinguishable from rheumatic disease [19].

Valvular disease is more likely to occur with greater radiation exposure, with the median interval between RT exposure and valvular disease being 23 years [8]. In thoracic cancer survivors treated with RT that develop radiation-induced severe aortic stenosis, TAVI should be taken into consideration even for those with intermediate surgical risk [56]. Still, Bouleti et al. found a nonsignificant increase in 5-year mortality in patients that developed radiation-induced aortic stenosis and were subsequently treated by TAVI [57]. Moreover, these patients have a higher risk of post-procedural atrial fibrillation and atrioventricular block requiring permanent cardiac pacing than the normal population [58]. Also, an echocardiographic study showed a higher incidence of paravalvular aortic regurgitation post-TAVI, probably due to the poor quality of the peri-valvular tissue that was affected by RT [59]. In patients with severe aortic stenosis undergoing SAVR, patients with prior mediastinal RT have significantly worse longer-term survival [60]. A study that was conducted at the Mayo Clinic and that compared TAVI and SAVR in patients previously exposed to RT showed a lower 30-day mortality with TAVI (1.8% versus 9.1%, *p* = 0.21) [61]. Surgical valve replacement of both the aortic and mitral valve of patients exposed to RT has been associated with dramatic decreases in 5-year postoperative survival [62,63]. To our knowledge, no large scale studies have been made to study solely the case of breast cancer survivors priorly exposed to RT.

c. **Heart failure and cardiomyopathies**

Breast cancer survivors who have undergone RT may experience heart failure with reduced or preserved ejection fraction due to various cardiomyopathies. In addition to ischemic etiologies, fibrosis of the myocardium can lead to decreased compliance and diastolic dysfunction, while hypertrophy and/or dilatation may result from valvular disease. Some patients may also develop restrictive cardiomyopathy as a result of constrictive pericarditis [64]. Interestingly, sometimes the right ventricle is more affected by the radiation, as it is located more anterior and closer to the chest wall [65]. Treatment of heart failure in those patients is guided the same way as in non-RT-exposed patients.

d. **Disease**

Pericardial damage after RT can take any form, from asymptomatic to acute pericarditis and cardiac tamponade or chronic constrictive pericarditis. A pathology post-mortem study showed that up to 70% of RT-exposed patients have some form of pericardial disease [66]. Currently, pericardial diseases are less common as a complication of chest radiotherapy, but are more likely to develop in patients treated with a radiation dose of at least 50 Gy. The incidence of pericarditis caused by radiation is closely linked to the dose received by the pericardium, with an increase from less than 5% to over 50% as the total radiation dose to the heart is increased from 40 to 50 Gy. Chronic pericarditis may occur from 6 months to 15 years after radiotherapy and can develop in up to 20% of patients treated with high radiation doses. The use of newer RT techniques that involve smaller total heart doses and conformational techniques has led to a decrease in these manifestations [67,68].

Pericardial effusion can develop as soon as a couple of days after radiation exposure, but cases have been reported when it appeared a month or even decades after initial treatment, and in some cases it remains chronic. The most serious form of pericardial involvement remains constrictive pericarditis, which usually develops 10 years after exposure [13]. Treatment is guided the same way as pericardial disease in non-RT-exposed patients, with the final solution being pericardiectomy in severe/recurrent forms. Cases of occult constrictive pericarditis have been reported 40 years after chest radiation exposure [69]. This highlights the importance of continuous monitorization of breast cancer survivors, even after long periods.

e. **Rhythm and conduction abnormalities**

Arrhythmias, conduction disease, and autonomic disease may appear. They may include AV block, bundle branch block, and sick sinus syndrome, and should be managed according to the 2021 ESC Guidelines on cardiac pacing and cardiac resynchronization therapy [2]. Any type of supraventricular arrhythmia may also arise acutely, of which AF is the most common [70].

One study that aimed to identify risk factors for arrhythmia occurrence after RT showed that whole heart dose, left ventricle, right ventricle and left atrium doses were not associated with an increased risk of arrhythmia (OR = 1.00, *p* > 0.90). In contrast, a non-significant trend toward a potentially higher risk of arrhythmia with increasing right atrium dose was observed (OR = 1.19, *p* = 0.60). This study also showed that patients with arrhythmia were more likely to have right-sided breast cancer than patients without arrhythmia [71]. To gain a more comprehensive understanding of the relationship between RT and the development of arrhythmias in breast cancer survivors, future studies should examine the potential role of right atrium exposure to RT. The laterality of breast cancer is also a crucial factor in the incidence of rhythm disturbances. Furthermore, in evaluating the impact of radiation on the heart, it is important to consider not only the whole heart dose but also the individual doses delivered to each of the four heart chambers. While the whole heart dose may be relatively low, one chamber of the heart may still receive a significant amount of radiation, making it essential to analyze radiation exposure in a more detailed and nuanced manner. Of note, in the role of arrhythmia development, the position of different structures in the heart should be noted, such as the sino-atrial node, which is located in the wall of the right atrium and the atrioventricular node, which is also located in the wall of the right atrium. For example, right bundle branch blocks are present more frequently, as a result of both myocardial fibrosis, as well as of direct exposure to radiation, due to their anterior position in the interventricular septum, which makes them very exposed to the radiation field [72]. Infranodal blocks occur more often than nodal blocks [12].

Studies have shown that supraventricular and ventricular arrhythmias are more common in patients after thoracic RT, a possible mechanism being the larger amount of myocardial fibrosis [73]. Atrial fibrillation (AF) has an increased incidence in cancer patients. The proposed mechanisms for the pathogenesis of AF in cancer patients (other than classic risk factors) are cancer and cancer-treatment-related factors, such as inflammation, fibrosis and hypercoagulability [74]. One study that analyzed a large cancer population found that patients who received radiation therapy had a significantly higher prevalence of AF compared to patients who did not receive radiation therapy (5.9 vs. 4.2%; *p* = 0.046) [75]. Moreover, a recent study that investigated the appearance of AF after RT for pulmonary cancer found an increased AF incidence in the first 30 days following RT [76]. The maximal dose delivered to the sino-atrial node in RT for lung cancer was an independent factor associated with the occurrence of AF [77]. In the case of breast cancer patients, the highest risk of AF was present in women who did not have surgery. The incidence of new onset AF was reported to be as high as 4% in breast cancer patients. These women had a three-fold higher risk of dying from heart or blood vessel problems within one year. Interestingly, women who received brachytherapy, in which radioactive seeds are placed in or near the tumor, had half the risk of developing AF than women who received external beam radiation [78]. It remains unclear whether the risk of AF is increased solely due to cancer itself or as a side effect of cancer therapy, or if cancer populations are inherently at a higher risk due to certain population characteristics or an increased incidence of comorbidities. In fact, comprehensive data on the real prevalence of cancer-therapy-induced AF are lacking.

A very recent study by Zafar et al. found that left atrial appendage (LAA) volume rather than left atrium (LA) volume (measured by 3D-volume-rendered cardiac CT) has a poor prognosis in cancer survivors treated with prior thoracic RT [79]. To our knowledge, no large population studies have been made regarding the predictive capacities of LA strain in breast cancer patients treated with RT.

f. **Effects on cardiac implantable devices**

The number of patients with cardiac implantable devices is rising, along with the number of cancer patients. It is therefore expected that a part of breast cancer patients that are being exposed to RT might have cardiac implantable devices. RT can lead to the malfunction of cardiac implantable electronic devices (CIEDs). The risk of CIED malfunction increases with higher radiation doses (with 2–5 Gy generally being considered the dose threshold) and with the use of high-energy photon RT. As a result, non-neutron-producing treatment is preferred for patients with a CIED. Malfunction of these devices can result in temporary or permanent damage to the device, leading to pacing issues. Patients with a CIED should be evaluated by their cardiologist or electrophysiologist according to group risk and task group paper recommendations. In some cases, relocating the CIED may be necessary, which carries additional risks [2,80].

Patients who require ventricular assist devices (VADs) are increasingly being diagnosed with neoplasia (7% of VAD patients) and may require radiotherapy. Therefore, close monitoring is essential. To detect VAD dysfunction, monitoring of vital signs is crucial. When treating these patients with radiotherapy, lower energies (<10 MV) and conformal radiation methods should be used, with the aim of minimizing the dose to the VAD components. However, there are currently no established protocols for these patients [81].

## 6. Cardiac Imaging Follow-Up in Patients Receiving RT for Breast Cancer

Echocardiography is recommended to screen for cardiac abnormalities 5–10 years after RT and every 5 years thereafter [19]. Patients at high risk of developing radiation-induced cardiac disease (such as those receiving high doses of radiation) may benefit from echocardiography as early as 6–24 months after receiving RT [19]. According to current ESC cardio-oncology guidelines, LVEF should be assessed routinely by 3D- or 2D-echocardiography before, during, and after cancer treatment [2]. The ASE/EACVI expert consensus on multimodality imaging in cancer patients states that left-ventricular GLS determined through Speckle Tracking echocardiography is superior to LVEF when trying to detect subclinical left-ventricle dysfunction in cancer patients [19]; so, the two methods should be combined in the assessment of patients with neoplasia.

As the most anterior cardiac chamber, the right ventricle is the first to be exposed to the radiation beam during treatment. Notably, while echocardiographic changes in right ventricle parameters have not been reported in patients with right-sided breast cancer who have undergone RT, such changes have been observed in patients with left-sided breast cancer who received RT. This may be attributed to both the right ventricle’s anterior position and the conformational fields used in treating left- versus right-sided breast cancer [82]. A study that investigated patients with left-sided breast cancer found no significant differences in classical parameters of the right ventricle before and 40 days after RT, with the exception of tricuspid annular plane systolic excursion—TAPSE—which was significantly reduced after RT [83]. Another study showed that 6 weeks after RT exposure, there was a small reduction in the global right ventricle strain and in the apical and the mid segments and that at 12 months there was a statistically significant GLS right-ventricle reduction [84]. Li J et al. showed that the Tei index of the right ventricle and tricuspid annular displacement can be used as markers of right heart injury after RT for thoracic tumors [85].

In right-sided cancer, strain and tissue Doppler seem to decrease in the basal anterior left ventricle myocardium, but global heart function remains unchanged, while for left sided-breast cancer, changes in the apical territory, as well as a decline in GLS have been found. Those changes appear to be mirrored in some cases by ECG changes in the same territories and by slight increases in hs-cTnI and NTproBNP [85].

One early study using strain analysis weekly in the first 6 weeks post-RT exposure found the lowest strain among patients was with the longest duration and highest dose of RT [86]. Similar data were confirmed by Chang et al., who showed that strain rate may reveal early RT-induced heart toxicity [87]. More recent information shows that decreased strain rates last up to 14 months after RT exposure, preponderant in the anterior and apical segments in left-sided breast cancer [88] and that GLS becomes stable at 24 months after RT [89].

The EARLY-HEART study proved a strong relationship between cardiac dose and the occurrence of subclinical left-ventricle dysfunction at 6 months post-RT, measured by strain analysis (>15% reduction in GLS) [90]. Another study showed that radiation dose is correlated with the subclinical left-ventricle segment strain analysis alteration [91]. Strain imaging is of great use in discovering subclinical cardiac toxicity in breast cancer patients exposed to RT and can have a high impact in the future management of the case.

In patients with poor acoustic windows or with high sensitivity of the skin (sometimes encountered after breast cancer surgery) cardiac MRI should be taken into account for evaluation of the heart chamber structure and function, as well as for valvular disease identification and quantification. Moreover, cardiac MRI is considered the gold-standard for left-ventricle ejection fraction quantification [92]. A recent study that investigated myocardial injury detected by T1 and T2 mapping on cardiac magnetic resonance showed that only epirubicin-based chemotherapy, but not left-sided radiotherapy, resulted in increased T1/T2 myocardial relaxation times (which signify myocardial oedema), as a marker of early myocardial injury [93].

## 7. Biomarkers

A number of studies have evaluated the predictive ability and sensitivity of various biomarkers in the occurrence of different cardiovascular manifestations in patients exposed to chest radiotherapy. The biomarkers studied include common biomarkers, such as N-terminal pro–B-type natriuretic peptide (NT-proBNP) and troponins, and a number of special biomarkers (placental growth factor (PlGF), growth differentiation factor (GDF)-15, TGF-β1, etc.) [94,95].

Demissei et al. found no statistical increase at 20 days post-RT exposure in NT-proBNP, high-sensitivity troponin T, PlGF, and GDF-15 [94].

Longer term follow-up (5 to 22 months after RT) studies found no changes in the levels of TnT but demonstrated increased levels of NT-proBNP in left-sided breast cancer patients (NT-proBNP median 90.0 pg/mL; range, 16.7–333.1 pg/mL after RT exposure, compared to NT-proBNP median 63.2 pg/mL; range, 11.0–172.5 pg/mL before RT exposure) [96]. Palumbo et al. reported BNP to have significant increased values (*p* < 0.001) at 1 month (mean 23.96 ± 15.08 pg/mL, range 5.14–68.83 pg/mL) and 6 months (mean 23.72 ± 15.32 pg/mL, range 5.28–60.46) after RT and to start decreasing after 12 months (mean 20.36 ± 16.16 pg/mL, range 1.57–63.61 pg/mL) [97].

To date, the only biomarkers used in current clinical practice and also included in the guidelines with recommendations to be measured at baseline, during and after treatment, in order to stratify the risk and prognosis, are cTnI/I, BNP and NTproBNP [2].

## 8. Conclusions and Future Directions

The incidence of breast cancer and CV disease co-occurrence is increasing annually. Fortunately, advances in treatment have made breast cancer a curable disease. Nevertheless, RT remains a key component of breast cancer treatment strategies, and although contemporary techniques have significantly reduced the risk of RT-induced cardiac dysfunction, cardiotoxicity remains a concern. Therefore, clinicians must continue to monitor and manage potential cardiovascular effects, particularly when deciding between mastectomy and less-invasive procedures. Additionally, radiation’s impact on different heart chambers must be taken into account for left- versus right-sided breast cancer patients. Long-term follow-up of RT-treated patients with clinical, biochemical, and multimodal imaging techniques is necessary to detect and treat cardiac damage at an early stage. For optimal patient management with maximal therapeutic effect and minimal cardiovascular toxicity, an interdisciplinary approach is recommended. Future studies should focus on the early prediction of subclinical cardiovascular toxicity to prevent its occurrence.

## Figures and Tables

**Table 1:** Risk factors for developing cardiovascular disease after radiotherapy.

Modifiable Risk Factors		Not Modifiable Risk Factors
Lifestyle risk factors	Smoking Sedentary High alcohol intake	Age: Older patients are generally at higher risk of CV disease, but also younger age at cancer diagnosis/treatment Pre-existing CV disease Diabetes Family history
CV risk factors	High cholesterol Obesity	
Technical factors	Radiation dose, volume, field Radiation technique	
Treatment	Previous/concomitant cancer treatment: chemotherapy, hormone therapy	

## Data Availability

Not applicable.

## Abbreviations

RT	radiotherapy
LVEF	left-ventricle ejection fraction
GLS	global longitudinal strain
LS	longitudinal strain
Gy	Gray
MHD	mean heart dose
CV	cardiovascular
CT	computed tomography
ECG	electrocardiogram
NT-proBNP	N-terminal-pro-brain natriuretic peptide
Tn	troponin
DIBH	deep-inspiration breathing hold
LAD	left descending artery
CAD	coronary artery disease
ACEi	angiotensin converting enzyme inhibitors
RCA	right coronary artery
OCT	optical coherence tomography
CAC	coronary artery calcium
PCI	percutaneous coronary intervention
SPECT	stress single-photon emission CT
PET	positron emission tomography
CMRI	cardiac magnetic resonance imaging
CCTA	coronary computed tomography angiography
TAVI	transcatheter aortic valve implantation
SAVR	surgical aortic valve replacement
AF	atrial fibrillation
CIEDs	cardiac implantable electronic devices
VADs	ventricular assist devices
TAPSE	tricuspid annular plane systolic excursion
PlGF	placental growth factor
(GDF)-15	growth differentiation factor
